# Non-invasive ventilation in neonates: a review of current literature

**DOI:** 10.3389/fped.2023.1248836

**Published:** 2023-11-28

**Authors:** Viraraghavan Vadakkencherry Ramaswamy, Risha Devi, Gunjana Kumar

**Affiliations:** ^1^ Department of Neonatology, Ankura Hospital for Women and Children, Hyderabad, India; ^2^ Department of Neonatology, Motherhood Hospital, Bengaluru, India; ^3^ Department of Neonatology, National Institute of Medical Sciences, Jaipur, India

**Keywords:** neonate, preterm, RDS, non-invasive ventilation, continuous positive airway pressure, high flow oxygen therapy, nasal intermittent positive airway pressure, nasal high frequency ventilation

## Abstract

Moving from an era of invasive ventilation to that of non-invasive respiratory support, various modalities have emerged resulting in improved neonatal outcomes. Respiratory distress is the commonest problem seen both in preterm and term neonates, and the use of appropriate respiratory support could be lifesaving. This article reviews the currently available non-invasive ventilation (NIV) strategies in neonates including nasal continuous positive airway pressure, nasal intermittent positive pressure ventilation (NIPPV), bi-level CPAP, heated humidified high flow nasal cannula, nasal high-frequency ventilation (NHFV) and non-invasive neutrally adjusted ventilatory assist (NIV-NAVA). Though multiple systematic reviews and meta-analyses have indicated the superiority of synchronized NIPPV over the other forms of non-invasive respiratory support in neonates, there is no single NIV modality that universally suits all. Hence, the choice of NIV for a neonate should be individualized based on its efficacy, the disease pathology, resource settings, the clinician's familiarity and parental values. Future studies should evaluate emerging modalities such as NIV-NAVA and NHFV in the respiratory management of neonates as the evidence pertaining to these is insufficient.

## Introduction

The incidence of preterm births has shown a steady increase over the past few decades globally (1). Since prematurity is one of the leading causes of neonatal mortality, this has had a significant impact on childhood mortality rates as it forms the major proportion of under-5 mortality, especially in low- and middle-income countries (LMICs) (2, 3). The occurrence of respiratory distress syndrome (RDS) is postulated to be around 12% among those born preterm. RDS is not only associated with significant short- and long-term morbidities but is also one of the commonest causes of mortality in preterm neonates (3). Moreover, mortality attributed to RDS is 10 times higher in LMICs when compared to high-income countries (4). Administration of antenatal corticosteroids, exogenous surfactant therapy and respiratory support (invasive or non-invasive) form the basis of prevention and treatment of RDS. Over the period of past few decades, invasive mechanical ventilation (IMV) has been supplanted by non-invasive ventilation (NIV) as IMV has been shown to be associated with major morbidities. One of the severe morbidities attributed to IMV is bronchopulmonary dysplasia (BPD) (5, 6). BPD itself has been associated with various adverse outcomes such as pulmonary hypertension, increased susceptibility to respiratory infections during infancy, neurodevelopmental impairment and cerebral palsy (7). Despite advances and improvements in IMV strategies, only volume-targeted ventilation (VTV) has been shown to reliably reduce the occurrence of BPD (8–10).

As early as 1987, Avery et al. had reported a reduced incidence of BPD with the use of nasal continuous positive airway pressure (CPAP) (11). Rapid strides in the field of NIV have happened since then (12). Currently, multiple NIV modalities are being used in treating respiratory distress in neonates. These include heated humidified high flow nasal cannula (HHHFNC), CPAP, bilevel CPAP (BiPAP), non-invasive positive pressure ventilation (NIPPV), nasal high-frequency ventilation (NHFV) and non-invasive ventilation with a neurally adjusted ventilatory assist (NIV-NAVA). These modalities vary with respect to many factors such as their mechanism of action, efficacy, safety cost and healthcare provider's familiarity with their use. Multiple systematic reviews and meta-analyses have been published on NIV and its various aspects in the recent years. The objective of our review was to summarize the findings of these systematic reviews so that it would aid the clinicians to interpret the evidence with ease and hence, guide safe clinical practice.

NIV includes respiratory support modalities that do not require the insertion of an endotracheal tube. The basic layout of the devices utilized for NIV consists of a source of oxygen and airflow, an air-oxygen blender, a servo-controlled humidifier and a nasal interface. Depending on the complexity of the device, a single or double-pressure generator, expiratory flow sensors and sensors for assessing diaphragmatic movement are its various components.

## CPAP

The earliest documented use of CPAP in preterm neonates for providing respiratory support was in 1971 by Gregory et al. (13). CPAP administered through nasal prongs or mask was the most prevalent form of non-invasive respiratory support during those times (14, 15). Subsequently, with the introduction and refinement of IMV in neonates, use of CPAP declined in the subsequent decade. This trend changed with the landmark publication by Avery et al. in 1987 (11). They reported the incidence of chronic lung disease (BPD) in 8 tertiary care neonatal intensive care units (NICUs) in North America, specifically to evaluate the likely causes for the difference in the incidence of BPD between these NICUs. The group found that the University of Columbia which used CPAP quite extensively with restricted IMV use had the lowest incidence of BPD. This reinvigorated the interest in CPAP globally, with its use being researched in various disease settings in neonates.

The mechanism of action of CPAP is multi-faceted as listed below:

• It improves the functional residual capacity (FRC) and hence decreases the work of breathing (16).
• It decreases intrapulmonary shunting (17).
• It stabilizes the compliant chest wall of preterm neonates and splints the airway (18, 19).
• It decreases thoracoabdominal asynchrony in the neonate (20).
• It aids in accelerating the growth of the immature lung and improves protein content per gram of the lung tissue (21).
• It reduces alveolar edema, conserves surfactant and decreases the occurrence of apnea, especially in preterm infants (22).

A recent systematic review of pre-clinical studies assessed the short- and long-term effects of CPAP use. This review reported that CPAP use in animal neonatal models resulted in improved ventilation and oxygenation (23). CPAP did not disrupt alveolar architecture or pulmonary microvasculature when compared to other non-ventilated or ventilated animal models. It was also shown that early weaning from IMV to CPAP prevents lung injury. Finally, CPAP not only improved the lung mechanics such as its compliance, it also increased the phosphatidylcholine levels which is essential for surfactant production (23).

### Generation and delivery of CPAP

It requires 3 major components: (a) flow generator, (b) positive pressure generator device and (c) airway interface.

#### Flow generation

A source of warm, humidified, blended air-oxygen mixture with an inline flow generator is present. The flow could be either constant or variable.

Constant flow devices provide a constant flow of gases as set by the clinician. These include the commonly used low-cost bubble CPAP and CPAP delivered through a ventilator. Variable flow devices provide a differential flow of incoming gases which is dependent on the phase of respiratory cycle of the spontaneously breathing neonate. Since this decreases the work of breathing, it has gained popularity since its introduction in 1988 (24). These devices use the Bernoulli principle and gas entrainment mechanisms to create a fluidic flip effect causing the change in direction of incoming gases during the expiratory phase of the neonate, and hence decrease the work of breathing (25, 26). There are several variable flow devices which are presently available for use.

#### Positive pressure delivery

The positive pressure delivered during CPAP may be generated through one of the following techniques:

• The expiratory valve of the ventilator which adjusts the expiratory pressure.
• Adjustment of the inspiratory flow through flow drivers or ventilators.
• The Bubble CPAP system produces a positive pressure by placing the far end of the expiratory tubing under water. The pressure is adjusted by altering the depth of the tube under the surface of the water.

Underwater bubble CPAP systems are most widely used globally, and more so in low-resource settings. The predominant reasons for their popularity are the low cost and maintenance, simplicity of use coupled with almost equal effectiveness as the other CPAP devices (27–31). Multiple studies have reported that bubble CPAP is as effective as or even better than ventilator-delivered CPAP for delivering effective positive pressure ventilation (27, 28).

A Cochrane review (27) published in 2023 investigated the efficacy and safety of bubble CPAP compared to other forms of CPAP namely, ventilator-driven and variable flow CPAP. The meta-analysis included 15 studies (1437 neonates) and reported that there was a reduced risk of treatment failure with bubble CPAP compared to the other forms [Risk Ration (RR) (95% confidence interval (CI)): 0.76 (0.60–0.95)]. The number needed to treat (NNT) for an additional beneficial outcome was 20. However, bubble CPAP was shown to be associated with an increased risk of nasal injury when compared to the other CPAP devices [RR (95% CI): 2.29 (1.37–3.82)]. There were no significant differences in the risk for mortality [RR (95% CI): 0.93 (0.64–1.36)], pneumothorax [RR (95% CI): 0.73 (0.40–1.34)] or BPD [RR (95% CI): 0.76 (0.53–1.10)].

Bharadwaj et al. (28) performed a meta-analysis (19 studies) to investigate the efficacy of bubble CPAP vs. other forms of CPAP devices in preterm neonates. The authors reported that the primary outcome of treatment failure was significantly lesser in neonates treated with bubble CPAP [RR (95% CI): 0.75 (0.57–0.98)]. There were no differences in the risk of mortality [RR (95% CI): 0.82 (0.47–1.42)], BPD [RR (95% CI): 0.8 (0.53–1.21)] and air leak [RR (95% CI): 0.80 (0.42–1.55)]. However, the risk of nasal injury was higher with bubble CPAP when compared to the other forms [RR (95% CI): 2.04 (1.33–3.140)].

A recent cross-over study evaluated the inspiratory efforts in preterm neonates when 3 different types of variable flow CPAP devices were used. This study did not find any difference between the devices (32).

#### Airway interface

Following nasal interfaces are used in neonates:

• Long nasal prongs/nasopharyngeal prongs.
• Short binasal prongs.
• Nasal masks.
• Nasal cannula with long and narrow tubing (CLNT) (RAM cannula, Neotech, Valencia) (33).

Traditionally, short binasal prongs used to be the most commonly used interface. This is due to their effectiveness, and hence had replaced the older interfaces namely, single nasal and nasopharyngeal prongs (34). Nasal masks are more contemporary in the evolution of CPAP interfaces, and are much smaller than face masks. Further, nasal masks have been associated with reduced risk of nasal trauma. An adequate seal avoiding leak at the nasal end is paramount for CPAP delivery, which was a cause of concern with the use of nasal masks.

The latest Cochrane review (2022) compared the use of nasal masks vs. nasal prongs for delivery of CPAP (12 studies, 1,604 neonates). The authors reported that the use of nasal masks was associated with lower treatment failure rates and decreased nasal injury (including moderate-severe injury). There was no difference in the outcomes of mortality, BPD or pneumothorax between the two groups (35) (Table 1). Similar results were reported by Razak et al. (36), King et al. (37) and Jasani et al. (38) in their respective meta-analyses. Moreover, King et al. also reported that there was a decreased risk of moderate to severe BPD and need for second dose of surfactant in the nasal mask group when compared to the short binasal prongs group (low to very low certainty of evidence) (37).

**Table 1:** Summary of recent meta-analyses comparing nasal mask and short binasal prongs for delivery of NCPAP.

Author year	Study population (*n*)	Intervention	Outcomes				
			Treatment failure (RR, 95% CI)	All-cause mortality (RR, 95% CI)	Pneumothorax (RR, 95% CI)	Nasal injury (RR, 95% CI)	BPD (RR, 95% CI)
Prakash et al. 2022 (35)	1,604 neonates 12 studies 26–34 w	Nasal mask vs. nasal prong for CPAP delivery	0.72 (0.58–0.90)^b^	0.83 (0.56–1.22)^b^	0.93 (0.45–1.93)	0.55 (0.44–0.71)	0.69 (0.46–1.03)
Razak et al. 2020 (36)	1,091 neonates 11 studies All GA (1 trial included <28 w GA)	Nasal mask vs. nasal prong for CPAP delivery	0.72 (0.58–0.90)^b^	0.85 (0.59–1.22)	1.03 (0.53–2.00)	0.64 (0.55–0.74)	0.84 (0.53–1.33)
King et al. 2019 (37)	665 neonates 7 studies 26 to <37 w	Nasal mask vs. nasal prong for CPAP delivery	0.72 (0.53–0.97)^b^	0.91 (0.59–1.38)	0.70 (0.27–1.82)	0.71 (0.59–0.85)^b^	0.94 (0.70–1.26)
Jasani et al. 2018 (38)	544 neonates 5 studies 26 to <37 w	Nasal mask vs. nasal prong for CPAP delivery	0.63 (0.45–0.88)^b^	—^a^	—^a^	0.41 (0.24–0.72)	—^a^

^a^ Risk ratio not mentioned in the paper but reported as no significant difference.

^b^ Primary outcomes; RR, risk ratio; CI, confidence interval; GA, gestational age; w, weeks; CPAP, continuous positive airway pressure; BPD, bronchopulmonary dysplasia.

### CPAP application in the Delivery room (DR)

Earlier, extremely preterm and very preterm neonates were routinely intubated, and given surfactant in the DR prophylactically. This was followed by a multitude of studies investigating the use of DR CPAP to avoid routine intubation in the DR. The current evidence suggests that early use of CPAP in the DR with selective use of early rescue surfactant using lesser invasive modalities is beneficial and preferable over prophylactic surfactant use followed by IMV in preterm neonates (39–45). CPAP provided in DR has also been found beneficial for late preterm and term neonates (46).

Subramaniam et al. (Cochrane 2021) (44) reported in a meta-analysis of 8 RCTs that prophylactic use of CPAP (application within 15 min of birth in DR) or very early CPAP (application within 60 min of birth) in preterm neonates led to decreased treatment failure (need for IMV) when compared to supportive treatment alone (supplemental oxygen) [RR (95% CI): 0.60 (0.49–0.74)]. However, the risk of BPD was similar between the two groups [RR (95% CI) 0.76 (0.51–1.14)]. The results were also comparable for the outcomes of mortality, BPD or mortality, pulmonary air leaks and intraventricular hemorrhage (IVH) grade >2. Use of prophylactic or very early CPAP was also compared to IMV with or without surfactant therapy. It was reported that the former decreased the risk of the combined outcome of BPD or death, BPD alone and failure of treatment with no effect on the outcomes of mortality, pulmonary air leak or IVH grade >2. The third comparison by the authors was between prophylactic and very early CPAP. The results were derived from a single RCT which reported no difference in mortality or BPD between the two groups. This meta-analysis included neonates of gestational age 24–32 weeks' gestation age (GA).

A network meta-analysis (NMA) published in 2022 compared the use of DR CPAP vs. other interventions. A trend towards decreased risk of IMV was indicated [RR (95% CrI) 0.75 (0.56–1.00)] (45). NMA is a statistical method to compare the efficacy and safety of multiple competing interventions in a single analysis. The final effect estimate is a combination of direct (from RCTs between two interventions) and indirect evidence (where RCTs between two interventions are not available, effect estimate being derived from other RCTs in the network). It provides more robust evidence than pairwise meta-analysis for comparisons where RCTs are available as indirect evidence is also added to the direct evidence from RCTs to derive the final NMA effect estimates. The NMA mentioned here included studies from LMICs only (7 RCTs, 4 observational studies; 4,210 neonates). The authors suggested future studies for evaluating the barriers in improving the effectiveness of DR CPAP in LMICs.

Another systematic review investigated the benefits of DR CPAP in late preterm and term neonates. The authors (2 RCT, 323 neonates) reported a significantly reduced need for admission to the NICU and need for respiratory support in the NICU. However, another systematic review including two observational studies enrolling 8,476 neonates reported that DR CPAP was associated with an increased risk of air leak syndromes when compared with no DR CPAP (46, 47).

### CPAP for RDS as primary respiratory support

CPAP is widely used as a primary respiratory support modality in neonates with RDS (48). CPAP for management of neonates with RDS should be initiated in the DR and continued while the neonate is being shifted to the NICU. Initiation of CPAP at birth in neonates who are relatively at higher risk of RDS (like those born prior to 30 weeks' GA) has been recommended by the European consensus guidelines for the management of RDS (49).

In a systematic review assessing the effect of early CPAP (initiated at the beginning of respiratory distress) compared to delayed CPAP (initiated when FiO_2_ was near 0.60), Ho et al. (48) found that there was no difference in the need for IMV [RR (95% CI): 0.77 (0.43–1.38)], mortality [RR (95% CI): 0.93 (0.43–2.03)], air leak [RR (95% CI): 1.09 (0.39–3.04)] and BPD [RR (95% CI): 1.42 (0.10–20.49)]. Only 4 RCTs (119 neonates, GA: 31–34 weeks) were included in the meta-analysis. These RCTs were performed in the 1970s and early 1980s, an era when both antenatal administration of steroids and respiratory management of preterm neonates were in their nascent stages.

Another Cochrane systematic review compared the initiation of CPAP with low pressure (5 cm H_2_O or less) vs. moderate to high pressure (>5 cm H_2_O) in both the settings of primary respiratory support and post-extubation support (50). For primary respiratory support, only 1 RCT with 271 preterm neonates was found to be eligible for inclusion. There was no significant difference in any of the outcomes namely, mortality or BPD [RR (95% CI): 1.02 (0.56–1.85)], mortality [RR (95% CI): 1.04 (0.51–2.12)], BPD [RR (95% CI): 0.80 (0.25–2.57)] or treatment failure [RR (95% CI): 1.00 (0.63–1.57)]. Two RCTs (117 neonates) were eligible for inclusion in the post-extubation setting. The results were quite similar with no significant difference in the composite outcome of mortality or BPD [RR (95% CI): 0.87 (0.51–1.49)], mortality [RR (95% CI): 2.94 (0.12–70.30)], BPD [RR (95% CI): 0.87 (0.51–1.49)] and treatment failure [RR (95% CI): 1.52 (0.92–2.50)] (50).

### CPAP for apnea of prematurity

CPAP has been reported to reduce the incidence of mixed and obstructive apnea in preterm neonates (51). It has also been proposed to reduce the frequency and duration of desaturation episodes in central apnea (52). An RCT conducted by Pantalitschka et al. (53) reported that variable flow devices are better than underwater bubble CPAP systems in reducing the risk of apnea of prematurity with median [Interquartile range (IQR)] event rates per hour being 2.8 (1.5–7.7) and 5.4 (3.0–9.8) in variable flow CPAP and constant flow bubble CPAP, respectively. A Cochrane systematic review to assess the efficacy of various CPAP devices in decreasing apnea of prematurity compared to supportive treatment or IMV is currently underway (54).

### CPAP for transient tachypnea of newborn (TTN)

CPAP helps in TTN by facilitating the clearance of lung fluid and helps to reduce the duration of NICU stay (55). A retrospective cohort study had evaluated the use of CPAP when compared to nasal cannula in late preterm and term neonates with TTN. The authors reported that after adjusting for birth weight and GA, CPAP decreased the maximal FiO_2_ requirement [Incidence rate ratio (IRR) (95% CI): 0.85 (0.76–0.96)] (56).

A recent Cochrane review assessed the efficacy of CPAP in TTN compared to other NIV modalities. Only 3 small RCTs were identified: CPAP vs. free flow oxygen, CPAP vs. NIPPV, and CPAP vs. NHFV (57). A meta-analysis was hence not feasible. The authors in their descriptive review concluded that CPAP decreased the duration of tachypnea compared to free flow oxygen [Mean Difference (MD) (95% CI): −21.10 h (−22.92 to −19.28); 1 RCT, 64 neonates)]. There were no differences for the outcomes of need for IMV [RR (95% CI): 0.30 (0.01–6.99)] and pneumothorax (none of the participants developed pneumothorax). NIPPV and CPAP (1 RCT, 40 neonates) were similar in their efficacy for the outcomes of requirement of IMV [RR (95% CI): 4.00 (0.49–32.72), pneumothorax [RR (95% CI): 1.00 (0.07–14.90)] and duration of tachypnea [MD (95% CI): 4.30 h (−19.14 to 27.74)]. The trial comparing NHFV to CPAP (1 study, 46 participants) reported that the duration of tachypnea was reduced in the NHFV group [MD (95% CI): −4.53 h (−5.64 to −3.42)].

Another RCT evaluated the benefit of prophylactic CPAP for 20 minutes in the DR following elective lower segment caesarean section in late preterm and term neonates. The comparator was standard care. The authors concluded that prophylactic CPAP significantly decreased the need for NICU admission (3% vs. 8.8%, *p* = 0.04) (58).

### CPAP for meconium aspiration syndrome (MAS)

CPAP may be of benefit in neonates with MAS. It possibly helps in maintaining FRC as alveoli are known to undergo atelectasis in MAS (59). A multi-centre RCT enrolling 135 neonates compared CPAP to standard care in neonates with moderate to severe respiratory distress due to MAS (60). The study reported that CPAP resulted in a decreased risk of IMV [3% vs. 25%, odds ratio (95% CI): 0.09 (0.02–0.43), *p* < 0.01].

### HHHFNC

HHHFNC works on the principle of providing inhaled gases at a flow higher than the neonate's innate inspiratory flow. This aids in reducing the physiological dead space in the upper airways. HHHFNC consists of 4 basic components: an air oxygen blender for titration of the fraction of inspired oxygen (FiO_2_) (61), a heating and humidification system which prevents upper airway damage by cold and dry gases, a high flow gas delivery system which delivers gases at flow rates of 2–8 litres/minute and a nasal cannula of varying sizes that occupy less than 50% of the neonates' nares.

The mechanism of action of HHHFNC is multi-factorial. Provision of continuous distending pressure which increases the FRC is one amongst them. It has been reported that distending pressure generated by HHHFNC might be similar to that generated by CPAP (62–67). A linear relationship between the flow rate and the distending pressure generated has been outlined in multiple studies (62, 66, 68). It has also been reported that a similar extent of distending pressure could be generated at relatively lower flow rates in preterm neonates (62, 65). Washout of upper airway dead space is another proposed mechanism of action of HHHFNC. Pre-clinical studies have shown that increased flow rates with HHHFNC result in lower carbon dioxide (CO_2_) levels (69, 70). Sivieri et al. in their in-vitro study reported better CO_2_ elimination with HHHFNC when compared to CPAP (70). Preterm neonates have an immature mucocilliary transport system which predisposes them to airway damage. The delivery of heated humidified gases not only protect their immature airway, but also decreases the energy expenditure (71, 72). All these result in decreased work of breathing (73).

### HHHFNC for DR stabilization

One in ten newly born infants requires respiratory assistance at birth (74). Various academic societies recommend DR CPAP in preterm neonates with respiratory distress (74, 75). HHHFNC has also been studied for respiratory stabilization in the DR.

A recently published RCT compared HHHFNC vs. CPAP for DR stabilization (76). Spontaneously breathing neonates of 28–36 weeks' GA were enrolled in the study (*n* = 124). Treatment failure was defined as the need for intubation or upgradation of support within the first 72 h. The authors reported that the non-inferiority of HHHFNC could not be conclusively proven as the 95% CI crossed both the line of no effect and the non-inferiority margin of 10%. There was no statistically significant difference in treatment failure rate in the HHHFNC group (13.1%) compared to the CPAP group (11.1%) [(Risk Difference (RD) (95% CI): 2% (−9.9% to 14.07%), *p* = 0.73)]. There were also no differences in any of the secondary outcomes of nasal injury, air leaks, time to treatment failure, duration of respiratory support and FiO_2_ requirement. It is to be noted that the study was underpowered to assess the primary outcome.

Another single-centre retrospective cohort study reported their experience of using HHHFNC for DR stabilisation over a period of 5 years (77). This study evaluated 292 neonates of less than 32 weeks' GA. The authors reported that 45% of these neonates required surfactant therapy. Also, more than three-fourths of these neonates either stayed on HHHFNC or required a lesser form of respiratory support. BPD developed in 36% of the survivors. A pilot study by Reynolds et al. (78) also reported the safe use of HHHFNC for DR stabilisation and transport in newly born preterm infants of less than 30 weeks' gestation. Further, 60% of these neonates did not require any upgradation of respiratory support in the first 72 h of life.

### HHHFNC for primary respiratory support

HHHFNC has been compared with CPAP as a primary respiratory support modality for preterm infants diagnosed with respiratory distress soon after birth. Several systematic reviews have been performed within the last 4 years with varying results (Table 1) (79–84). Most of these systematic reviews included preterm neonates of <37 weeks' GA. Only a limited number of studies enrolled extremely low gestational age neonates' (ELGANs) of less than 28 weeks' GA. The primary outcomes evaluated in most of these systematic reviews were quite variable. The Cochrane review reported on the outcomes of risk of mortality or BPD, mortality, BPD, treatment failure within the first 72 h and the need for IMV (80). The authors found that the use of HHHFNC as a primary respiratory support did not result in any significant difference in most of the outcomes except for a higher treatment failure rate in the HHHFNC group when compared to the CPAP group. Also, HHHFNC was associated with a decreased risk of nasal injury when compared to CPAP (Table 2). The review also compared the use of HHHFNC vs. NIPPV for primary respiratory support in preterm neonates. The authors reported there was no statistically significant difference between the two groups for the combined outcome of mortality or BPD, mortality, treatment failure, need for IMV and occurrence of pneumothorax. A similar finding of reduced risk of nasal injury in the HHHFNC group was also reported when compared to NIPPV [RR (95% CI): 0.21(0.09–0.47)].

**Table 2:** Summary of recent meta-analyses comparing HHHFNC and CPAP as primary respiratory support modality.

Author year	Study population (*n*)	Intervention (number of studies)	Outcomes						
			Mortality or BPD (RR, 95% CI)	Mortality (RR, 95% CI)	BPD (RR, 95% CI)	Treatment failure at 72 h (RR, 95% CI)	Need for mechanical ventilation (RR, 95% CI)	Pneumothorax/Air leak syndromes (RR, 95% CI)	Nasal injury (RR, 95% CI)
Hodgson et al. 2023 (80)	2,540 neonates 13 studies. <37 w	a) HHHFNC vs. CPAP (11) b) HHHFNC vs. NIPPV (4)	1.09 (0.74–1.60)^a^ 0.64 (0.30–1.37)^a^	0.78 (0.44–1.39)^a^ 0.78 (0.36–1.69)^a^	1.14 (0.74–1.76)^a^ —	1.70 (1.41–2.06)^a^ 1.27 (0.90–1.79)^a^	1.04 (0.82–1.31)^a^ 0.91 (0.62–1.33)^a^	0.66 (0.40–1.08) 0.78 (0.40–1.53)	0.49 (0.36–0.68) 0.21 (0.09–0.47)
Luo et al. 2022 (79)	3,351 neonates 27 studies <37 w	HHHFNC vs. CPAP (27)	—	—	—	1.17 (0.88–1.56)^a^	1.00 (0.84–1.19)^a^	0.65 (0.46–0.92)	0.36 (0.29–0.45)
Bruet et al. 2022 (82)	1,830 neonates 10 studies <37 w	HHHFNC vs. CPAP (10)	—	—	—	1.34 (1.01–1.68)^a^	0.90 (0.66–1.15)	—	0.48 (0.31–0.65)
Hong et al. 2021 (81)	2,886 neonates 21 studies <37 w	HHHFNC vs. CPAP (21)	—	0.96 (0.44–2.11)	1.11 (0.82–1.50)	1.03 (0.79–1.33)^a^		0.53 (0.33–0.86)	0.58 (0.50–0.68)
Brito et al. 2021 (83)	2,038 neonates 15 studies <37 w	HHHFNC vs. CPAP (15)	—	—	1.10 (0.90–1.34)^a^	—	—	1.06 (0.52–2.14)	2.00 (0.64–6.25)
Fleeman et al. 2019 (84)	1,600 neonates 9 studies <37 w	HHHFNC vs. CPAP (9)	—	1.03 (0.32–3.33)	1.14 (0.75–1.75)		1.15 (0.87–1.52)^a^	0.88 (0.46–1.67)	0.52 (0.37–0.74)

Data for use of HHHFNC in extremely low gestational age neonates is lacking.

^a^ Primary outcomes; RR, risk ratio; CI, confidence interval; w, weeks; HHHFNC, Heated humidified high flow nasal cannula; CPAP, continuous positive airway pressure.

The systematic review by Luo et al. (79), included an extensive literature search from Chinese databases. This systematic review included a total of 27 studies enrolling 3,351 preterm neonates. The authors reported that HHHFNC use was associated with similar rates of treatment failure and the need for IMV when compared to CPAP (Table 2). HHHFNC also resulted in a lower risk of air leak syndromes, nasal injury, decreased duration of oxygen support and earlier initiation of enteral feeding. A sensitivity analysis was performed after the exclusion of studies with a high risk of bias. The results pertaining to the outcome of decreased duration of oxygen support and earlier initiation of enteral feeding were contradictory. Bruet et al. (82), in their systematic review including 10 studies reported a higher risk of treatment failure with HHHFNC use when compared to CPAP when used as primary support. However, the need for IMV was similar. Further, the authors reported a lower risk of nasal injury with HHHFNC (Table 2). The authors also performed meta-regressions and did not find any significant effects of gestational age, birth weight, flow rates used for HHHFNC, or the type of CPAP devices used on the outcomes.

In conclusion, the use of HHHFNC as primary support in infants with ≥28 weeks GA may not result in any difference in the combined outcome of mortality or BPD when compared to CPAP. However, HHHFNC might be associated with an increased risk of need for upgradation of respiratory support within 72 h of initiation. There is no robust evidence for the use of HHHFNC as primary respiratory support in ELGANs.

### HHHFNC for post extubation support

HHHFNC has also been used as a weaning modality after extubation from IMV and its efficacy has been compared with other modes of NIV (81, 83–87). The recently published meta-analysis by Martins et al. (85) (7 studies, 1,044 infants) compared HHHFNC vs. CPAP. The primary outcomes were extubation failure rates at 72 h and 7 days. They reported no significant difference in the primary outcomes. The other outcomes of mortality, BPD, air leak syndrome, pneumothorax and abdominal distension were also similar between the groups (Table 3). As observed in the other systematic reviews on primary respiratory support, HHHFNC was associated with a decreased risk of nasal injury. It is to be noted that the studies including both preterm and term infants were evaluated in this review. An insignificant difference in extubation failure rate has been reported by Hong et al. (81) in their meta-analysis as well (10 studies, 1,378 neonates). It was also reported that HHHFNC was associated with significantly lesser nasal injury. Further, fewer patients developed pneumothorax in the HHHFNC group when compared to CPAP. In conclusion, most of the meta-analysis conducted in the last 5 years suggest no difference in extubation failure rates, mortality or BPD. A decreased risk of nasal injury with HHHFNC as a post-extubation respiratory support modality when compared to CPAP has been reported by most of these meta-analyses (Table 3). Likewise for primary respiratory support, the use of HHHFNC as a post-extubation modality requires further trials in the sub-group of ELGANs.

**Table 3:** Summary of recent meta-analyses comparing HHHFNC vs. CPAP as a respiratory support modality in the post-extubation setting.

Author year	Study population (*n*)	Intervention	Outcomes					
			Extubation failure at 72 h (RR, 95% CI)	Extubation failure at 7 days (RR, 95% CI)	BPD (RR, 95% CI)	Mortality (RR, 95% CI)	Air leak or Pneumothorax (RR, 95% CI)	Nasal Injury (RR, 95% CI)
Martins et al. 2022 (84)	1,044 neonates 7 studies All GA included	HHHFNC vs. CPAP	1.33 (0.67–2.63)^b^	1.18 (0.73–1.89)^b^	1.25 (0.59–2.65)	0.83 (0.45–1.53)	0.24 (0.03–2.25) 0.81 (0.23–2.86)	0.21 (0.08–0.52)
Hong et al. 2021 (81)	1,378 neonates 10 studies <37 w	HHHFNC vs. CPAP	—	1.23 (1.01–1.50)^b^	0.87 (0.71–1.07)	0.84 (0.50–1.43)	— 0.34 (0.12–0.91)	0.64 (0.52–0.78)
Brito et al. 2021 (83)	1,064 neonates 6 studies <37 w	HHHFNC vs. CPAP	—	—	1.08 (0.87–1.34)^b^	—	— —	—
Junior et al. 2020 (86)	645 neonates 4 studies <37 w	HHHFNC vs. CPAP	9% (−1% to 13%)^a^^,^^b^	—	0.81 (0.57–1.16)	—	— 0.33 (0.05–2.11)	0.21 (0.13–0.35)
Fleeman et al. 2019 (84)	1,201 neonates 10 studies <37 w	HHHFNC vs. CPAP	1.24 (0.81–1.89)^b^	0.84 (0.63–1.12)^b^	0.86 (0.70–1.06)	0.71 (0.31–1.60)	0.29 (0.11–0.76)	0.35 (0.27–0.46)
Wilkinson et al. 2016 (87)	934 neonates 6 studies <37 w	HHHFNC vs. CPAP	1.21 (0.95–1.55)	—	0.96 (0.78–1.18)^b^	0.77 (0.43–1.36)^b^	— 0.35 (0.11–1.06)	0.64 (0.51–0.79)

Data for use of HHHFNC in extremely low gestational age neonates is lacking.

^a^ Risk difference (95% CI).

^b^ Primary outcomes; RR, risk ratio; CI, confidence interval; GA, gestational age; w, weeks; HHHFNC, Heated humidified high flow nasal cannula; CPAP, nasal continuous positive airway pressure; BPD, bronchopulmonary dysplasia.

### HHHFNC as a weaning mode from other NIV modalities

There is no consensus on the timing or method of weaning from various NIV respiratory support modalities (88–90). Various methods like abrupt stoppage of CPAP, gradual lowering of PEEP, intermediate stoppage of CPAP for some time followed by re-application, weaning to HHHFNC, weaning to low flow nasal cannula or head box oxygen have been tried. But none of the strategies have been conclusively shown to be better (41). A recent meta-analysis of 15 trials enrolling 1,547 neonates assessed the various weaning strategies from NIV in preterm neonates (91). The primary outcomes of this systematic review were successful weaning at the first attempt and risk of weaning failure. It was reported that there was no significant difference in successful weaning at the first attempt when a gradual decrease of pressure was used in comparison to sudden discontinuation of CPAP [2 studies, 422 neonates, RR (95% CI): 1.30 (0.93–1.83)]. The postmenstrual age at which successful weaning was achieved was approximately 3 weeks lesser when HHHFNC was used for weaning from CPAP compared to sudden discontinuation of CPAP [MD (95% CI): −2.7 weeks (−3.87 to −1.52)]. However, there was no significant difference in weaning failure rate at first attempt between the step-down strategy and the abrupt stopping of CPAP [4 trials, 327 infants; RR (95% CI): 1.25 (0.79–1.97)]. None of the weaning strategies had any bearing on the incidence of BPD or length of hospital stay (91).

### HHHFNC during endotracheal intubation

Intubation of a hypoxemic neonate is often associated with adverse life-threatening events. The use of HHHFNC during endotracheal intubation has been compared to standard care (no nasal high flow or use of supplemental oxygen) in a recently published RCT (92). With the data derived from 251 intubations in 202 infants, the likelihood of successful intubation on the first attempt without physiological instability was significantly higher in the HHHFNC group compared to standard one (50.0% vs. 31.5%, adjusted RD, 17.6 percentage points; 95% CI, 6.0–29.2) with the number needed to treat (NNT) being 6 (95% CI, 4–17) (92).

### BiPAP

In this form of non-invasive respiratory support, a low and a high pressure is delivered to the neonatal airway at predefined rates through a nasal interface. The difference between the two pressure levels is usually 4 cm of H_2_O or less. The rate of delivery of the higher pressure range between 10 and 30 per minute, and the inspiratory time (Ti) is set between 0.5–1 s (93). This device needs an added pressure level as well as cycling of pressures at predefined times. There are specific machines that deliver BiPAP alone. Some ventilators also have the provision for BiPAP. Most machines that provide BiPAP can deliver a maximum positive inspiratory pressure of 15 cm H_2_O. BiPAP may provide an added benefit over CPAP by delivering a higher mean airway pressure. This might translate to better recruitment of alveoli. However, this is limited by the lack of appropriate measures for synchronisation with the neonate's spontaneous breathing efforts.

Recently, a meta-analysis was performed to compare the efficacy of BiPAP with CPAP. It was found that there was no significant difference in the duration of IMV [MD (95% CI): 0.04 days (−0.31 to 0.39)], incidence of BPD [RR (95% CI): 0.98 (0.60–1.59)], extubation failure or death [RR (95% CI): 1.00 (0.81–1.23)], death [RR (95% CI): 0.62 (0.15–2.48)] (94). This meta-analysis included 4 RCTs which had predominantly included preterm neonates born between 28 and 34 weeks' GA. An RCT comparing BiPAP with CPAP for management of RDS enrolled 85 very low birth weight (VLBW) neonates. This trial reported a reduction in treatment failure rates (4.4% vs. 22.5%) and apnea episodes (13.3% vs. 32.5%) with BiPAP use (95).

Another RCT which enrolled 540 neonates of less than 30 weeks' GA compared BiPAP vs. CPAP for post-extubation support. The authors reported similar outcomes for treatment failure [21% vs. 20%, adjusted odds ratio (95% CI): 1.01 (0.65–1.56), *p* = 0.97] (96).

### NIPPV

This form of non-invasive ventilation involves delivery of varying inspiratory and positive end-expiratory pressures (PEEP) with time cycling at pre-defined rates. The major differences from BiPAP are that the difference between peak inspiratory pressure (PIP) and PEEP can be above 4–5 cm of H_2_O, it utilises lower inspiratory times, higher respiratory rates, and can be synchronised or non-synchronised with the neonate's spontaneous respiratory effort (97).

NIPPV functions through multiple physiological mechanisms. It provides PEEP similar to CPAP leading to all the benefits ascertained to CPAP. In addition, it delivers PIP which is not provided by CPAP. This results in a higher mean airway pressure than that achieved with CPAP leading to better recruitment of alveoli (96). This also helps in better CO_2_ removal. The two major forms of NIPPV available are synchronised NIPPV (sNIPPV) and non-synchronised NIPPV (nsNIPPV). A post-extubation study among VLBW neonates had demonstrated decreased inspiratory work of breathing as monitored by esophageal manometry. Further, an improved tidal volume delivery was demonstrated with the use of sNIPPV when compared to CPAP (98). This study also reported improved minute ventilation at lower respiratory rates with sNIPPV use. NIPPV also improves FRC, reduces bradycardia and apnea events (99). Head's paradoxical reflex is considered to be an important physiological mechanism in NIPPV (100). This is an increase in inspiratory reflex in response to inflation of the pharynx by the pressure delivered during NIPPV (99, 100).

Delivery of non-synchronised breaths is bound to cause discomfort to neonates. Few studies have shown that improved ventilation occurs only with the synchronised breaths delivered during nsNIPPV (98, 99, 101, 102). Four types of triggering devices are used for achieving synchronisation in NIPPV. These are the abdominal capsule (Graseby capsule), pressure trigger device, flow trigger device and NAVA trigger device. The abdominal capsule detects the spontaneous effort by sensing abdominal excursion resulting in a delayed response, and hence causing inappropriate synchronisation (especially in neonates with tachypnea) (103, 104). Pressure trigger devices cannot be used for preterm neonates due to their poor inspiratory drive. A pressure trigger device delivers breath In response to decrease in pressure that occurs with a patient's inspiratory effort (101). A flow trigger is ubiquitous in neonatal ventilators for delivery of IMV. But the same flow sensor cannot be used for NIPPV as these circuits have significant leaks at the interface or when the neonate opens the mouth. A pneumotachograph provides a solution to this problem. When it is attached to a software it helps to detect a sudden change in flow with inspiratory effort, and hence circumvents the gradual continuous change in flow caused by leaks in the system (105). This device has been shown to be effective in reducing the need for intubation in neonates (106, 107). The most recent advancement in the field of NIV has been NAVA which synchronises delivered breaths to the electrical signal attained from the neonate's diaphragm making the synchronisation close to the ideal (108). This is made possible by the presence of sensors in a nasogastric tube inserted specifically for this purpose. These devices have also been shown to be effective in decreasing the risk of intubation and extubation failure in preterm neonates with RDS (109).

Interfaces for NIPPV are similar to those used with CPAP (100). A recently conducted non-inferiority trial reported that CLNT (RAM cannula, Neotech^,^ Valencia) was comparable to short binasal prongs for delivering NIPPV in preterm neonates of 24 to 33 weeks' GA (110). 166 preterm neonates were randomized in the study and 14% neonates in CLNT group required intubation within 72 h as compared to 18% in the short binasal prongs group [RD (95% CI): −3.6% (−14.8 to 7.6), within the non-inferiority margin]. The incidence of moderate to severe nasal injury was lower in the CLNT group (5% vs. 17%, *p* = 0.01), but there was no difference in the other outcomes. On the other hand, another RCT randomized 126 preterm neonates to receive NIPPV by short binasal prongs and CLNT (RAM cannula, Neotech^,^ Valencia), and reported a higher need for IMV (32.8% vs. 9.6%, *p* = 0.002) and surfactant administration (42.1% vs. 19.3%, *p* = 0.07) in the CLNT group (111). A meta-analysis (3 RCTs and 3 observational studies) stated that clinical benefit or harm could not be ruled out for the outcome of the need for IMV for CLNT (RAM cannula, Neotech^,^ Valencia) vs. short binasal prongs or nasal mask [RR (95% CI): 1.37 (0.61–3.04)] (112). The final verdict on whether one is better than the other still awaits us as more RCTs are needed to compare the two in various disease settings.

### NIPPV as primary respiratory support for RDS

A recent NMA comparing different methods of NIV in preterm neonates for primary respiratory support for RDS assessed 35 studies enrolling 4,078 preterm neonates with a mean GA of 31 weeks (113). NIPPV was found to be more likely to prevent treatment failure and decrease the need for IMV when compared to HHHFNC and CPAP, and was comparable to BiPAP in its efficacy (Table 4). The combined outcome of death or BPD was significantly lower with NIPPV use compared to CPAP. NIPPV was also associated with decreased incidence of air leaks compared to CPAP and BiPAP. The authors also reported that sNIPPV, nsNIPPV and BiPAP individually also decreased the need for IMV [RR (95% CrI): 0.54 (0.32–0.90), 0.61 (0.43–0.83), 0.51 (0.29–0.85)] and treatment failure when compared to CPAP (113). The authors however did not differentiate between the various forms of CPAP delivery systems. Further, a subgroup analysis for ELGANs was not performed.

**Table 4:** Summary of recent meta-analyses comparing NIPPV to other non-invasive ventilation techniques as primary respiratory support modality.

Author year	Study population (*n*)	Intervention (number of studies)	Outcomes						
			Mortality or BPD (RR, 95% CrI/CI)	Mortality (RR, 95% CrI/CI)	BPD (RR, 95% CrI/CI)	Treatment failure at 72 h (RR, 95% CrI/CI)	Need for mechanical ventilation (RR, 95% CrI/CI)	Air leaks (RR, 95% CrI/CI)	Nasal injury (RR, 95% CrI/CI)
Ramaswamy et al. 2020^c^ (113)	4,078 neonates 35 studies 24 to <37 w	NIPPV vs. HHHFNC	0.76 (0.41–1.42)	1.26 (0.42–4.11)	0.80 (0.40–1.52)	0.42 (0.30–0.63)^b^	0.65 (0.43–0.96)^b^	0.86 (0.40–1.93)	1.13 (0.06–24.87)
		NIPPV vs. CPAP	0.74 (0.52–0.98)	0.60 (0.37–0.89)	0.75 (0.48–1.09)	0.56 (0.44–0.71)^b^	0.60 (0.44–0.77)^b^	0.54 (0.30–0.87)	0.17 (0.00–2.13)
		NIPPV vs. BiPAP	0.78 (0.49–1.17)	0.48 (0.22–0.95)	0.72 (0.40–1.19)	0.81 (0.56–1.15)^b^	0.77 (0.41–1.21)^b^	0.36 (0.16–0.73)	—
Lemyre et al. 2016 (114)	1,885 neonates 10 studies 24–36 w	NIPPV vs. CPAP (10)	—	0.77 (0.51–1.15)	0.78 (0.58–1.06)	0.65 (0.51–0.82)^b^^,^^d^	0.78 (0.64–0.94)^b^	0.79 (0.42–1.48)	—
		Ventilator-NIPPV vs. CPAP (6)	—	0.80 (0.52–1.23)	0.73 (0.47–1.15)	0.63 (0.47–0.86)^b^^,^^d^	−0.08 (−0.14 to −0.02)^a^	0.49 (0.21–1.11)	—
		sNIPPV vs. CPAP (4)	—	0.25 (0.03–2.19)	0.43 (0.18–1.01)	0.65 (0.41–1.02)^b^^,^^d^	0.67 (0.42–1.06)	0.86 (0.30–2.43)	—
		nsNIPPV vs. CPAP (5)	—	0.83 (0.54–1.27)	0.74 (0.51–1.08)	0.60 (0.44–0.83)^b^^,^^d^	0.74 (0.60–0.92)	0.59 (0.24–1.40)	—

w, weeks; HHHFNC, Heated humidified high flow nasal cannula; CPAP, nasal continuous positive airway pressure; NIPPV, non-invasive positive pressure ventilation; sNIPPV, synchronized non-invasive positive pressure ventilation; nsNIPPV, non-synchronized non-invasive positive pressure ventilation; BPD, bronchopulmonary dysplasia.

^a^ Mean difference (95% Confidence interval).

^b^ Primary outcomes; RR, risk ratio; CI, confidence interval.

^c^ Network meta-analysis.

^d^ Outcome parameter was treatment failure within first 7 days of life.

An earlier Cochrane meta-analysis had reported outcomes while comparing synchronised, unsynchronised and ventilator-driven NIPPV with CPAP (114). The review included 10 studies and 1,885 preterm neonates (Table 4). The results of this meta-analysis also showed that NIPPV decreased the treatment failure rates and the need for IMV when compared to CPAP. An unexpected finding in the meta-analysis was the reduced treatment failure and IMV rate with nsNIPPV compared to CPAP, but not with sNIPPV. Other outcomes such as mortality, BPD and air leaks were similar. Benefit of NIPPV over CPAP for the subgroup of ELGANs could not be determined by the authors.

### NIPPV for post extubation support

Another NMA by Ramaswamy et al. compared various NIV modalities for post-extubation respiratory support (115). The NMA included 31 studies enrolling 3,899 neonates. The authors reported that the primary outcome of requirement for re-intubation was decreased with the use of nsNIPPV, sNIPPV, NHFV and variable flow CPAP (VFCPAP) when compared to constant flow CPAP (CFCPAP) [RR (95% CrI): 0.44 (0.27–0.67), 0.22 (0.12–0.35), 0.42 (0.18–0.81), 0.73 (0.52–0.99)]. Both sNIPPV and nsNIPPV were better than BiPAP in decreasing the risk of the primary outcome. sNIPPV was ranked as the best intervention as per the surface under the cumulative ranking (SUCRA) values. The authors also reported that nsNIPPV decreased re-intubation rates when compared to VFCPAP and HFNC. sNIPPV also reduced the risk of BPD and air leak compared to nsNIPPV, VFCPAP and CFCPAP. A Cochrane meta-analysis (2017) reported a reduction in the incidence of mortality, respiratory failure and the need for IMV with the use of NIPPV when compared to CPAP (116) (Table 5). Both these reviews did not analyse the ELGAN sub-group separately.

**Table 5:** Summary of recent meta-analysis comparing NIPPV to other non-invasive ventilation techniques for post extubation support.

Author year	Study population	Intervention (number of studies)	Outcomes					
			Mortality (RR, 95% CrI/CI)	BPD (RR, 95% CrI/CI)	Respiratory failure post extubation (RR, 95% CrI/CI)	Need for mechanical ventilation (RR, 95% CrI/CI)	Air leaks (RR, 95% CrI/CI)	Nasal injury (RR, 95% CrI/CI)
Ramaswamy et al. 2020^b^ (115)	3,899 neonates 31 studies 24 to <37 w	sNIPPV vs. HFNC	—	0.72 (0.37–1.21)	—	0.24 (0.12–0.41)^a^	1.57 (0.18–5.81)	—
		nsNIPPV vs. HFNC	—	2.50 (0.85–5.99)	—	0.49 (0.27–0.80)^a^	1.71 (0.19–6.39)	4.96 (0.44–19.62)
		sNIPPV vs. CFCPAP	—	0.65 (0.38–0.98)	—	0.22 (0.12–0.35)^a^	0.36 (0.07–0.96)	—
		nsNIPPV vs. CFCPAP	—	2.26 (0.79–5.46)	—	0.44 (0.27–0.67)^a^	0.41 (0.06–1.25)	1.48 (0.16–5.41)
		sNIPPV vs. VFCPAP	—	0.52 (0.25–0.92)	—	0.30 (0.16–0.50)^a^	0.70 (0.13–1.19)	—
		nsNIPPV vs. VFCPAP	—	1.71 (0.69–3.72)	—	0.61 (0.36–0.97)^a^	0.75 (0.15–2.24)	3.94 (0.27–15.67)
		sNIPPV vs. BiPAP	—	0.56 (0.24–1.03)	—	0.32 (0.14–0.64)^a^	0.33 (0.02–1.29)	—
		nsNIPPV vs. BiPAP	—	1.84 (0.67–4.18)	—	0.66 (0.30–1.37)^a^	0.31 (0.03–1.04)	9.45 (0.31–36.34)
		sNIPPV vs. NHFOV	—	1.10 (0.37–2.51)	—	0.59 (0.21–1.30)^a^	—	—
		nsNIPPV vs. NHFOV	—	3.85 (0.89–11.32)	—	1.19 (0.46–2.58)^a^	—	—
Lemyre et al. 2017 (116)	1,431 neonates 10 studies 24 to <37 w	NIPPV vs. CPAP (10)	0.68 (0.49–0.99)	0.94 (0.80–1.10)	0.70 (0.60–0.80)	0.76 (0.65–0.88)^a^	0.48 (0.28–0.82)	—
		sNIPPV vs. CPAP (5)	0.97 (0.21–4.44)	0.64 (0.44–0.95)	0.25 (0.15–0.41)	0.33 (0.19–0.57)^a^	0.35 (0.14–0.90)	—
		nsNIPPV vs. CPAP (4)	0.35 (0.16–0.75)	0.74 (0.47–1.16)	0.65 (0.46–0.93)	0.65 (0.46–0.93)^a^	1.10 (0.58–2.08)	—

—, Comparison not done for this outcome; w, weeks; HHHFNC, Heated humidified high flow nasal cannula; CPAP, nasal continuous positive airway pressure; CFCPAP, continuous flow nasal continuous positive airway pressure; VFCPAP, variable flow nasal continuous positive airway pressure; NIPPV, non-invasive positive pressure ventilation; sNIPPV, synchronized non-invasive positive pressure ventilation; nsNIPPV, non-synchronized non-invasive positive pressure ventilation; BPD, bronchopulmonary dysplasia.

^a^ Primary outcomes; RR, risk ratio; CI, confidence interval.

^b^ Network meta-analysis.

## Non-invasive ventilation with neurally adjusted ventilatory assist (NIV-NAVA)

As described earlier, NAVA is a technique for achieving synchronisation between the ventilator breaths and neonatal spontaneous effort by assessing the electrical activity of diaphragm (EAdi). This helps in overcoming the major challenges with the synchronisation of NIV in neonates. The synchronisation is expected to be near ideal as both the diaphragm and the respiratory support device are acting upon the same electrical signal reaching the diaphragm (117). NIV-NAVA has been shown to reduce the PIP requirement and improve oxygenation in preterm neonates (118, 119). Firestone et al. reported in their retrospective study that NIV-NAVA reduces the risk of apnea when compared to CPAP (109). Another retrospective study reported a reduction in extubation failure rates with NIV-NAVA when compared to CPAP in preterm neonates (6.3% vs. 37.5%, *p* = 0.041) (120). The nasal interfaces used with NAVA are similar to those used with other forms of NIV with the addition of a nasogastric tube housing the electrodes for assessing EAdi.

Commonly used initial settings for NIV-NAVA include an appropriate level of PEEP, a PIP which is 5–8 cm H_2_O above the set PEEP, and a predefined respiratory rate which is set to provide ventilation to the neonate as a backup for apnea (121). The most commonly used EAdi trigger level to initiate a breath is 0.5 microvolt.

Multiple observational studies and RCTs have been conducted comparing NIV-NAVA with other modes of NIV. A retrospective review studying ELGANs showed that NIV-NAVA can be safely used for post-extubation support (74% neonates extubated from IMV after surfactant administration did not require reintubation) (122). A Cochrane meta-analysis (2020) compared the efficacy of NIV-NAVA with other types of non-invasive respiratory support in neonates (123). Only 2 RCTs (23 neonates, >28 weeks' GA) were eligible for inclusion. 1 RCT (16 neonates) reported no significant difference in treatment failure rate between NIV- NAVA and NIPPV [RR (95% CI): 0.33 (0.02–7.14)]. NIV-NAVA was also shown to be associated with significantly increased respiratory rate [MD (95% CI): 7.22 breaths/minute (0.21–14.22)] compared to NIPPV. However, the maximum EAdi signal was similar in both the groups [MD (95% CI): −1.75 microvolt (−3.75 to 0.26)].

## Nasal high-frequency ventilation (NHFV)

Incidence of BPD has stayed stable despite rapid advancements in treatment strategies for preterm neonates such as the use of non-invasive support, DR CPAP, early selective surfactant with less invasive surfactant administration technique, avoidance of IMV, use of volume-targeted ventilation and timely administration of medications like corticosteroids and caffeine (124, 125). Most likely cause for this appears to be the increased survival of ELGANs. Hence, there has been a continual search for other provisions that could decrease the incidence of BPD. Amongst the non-invasive respiratory support strategies, NIV-NAVA and NHFV are the most focused ones as of present. NFHV is expected to negate the effect of patient-ventilator asynchrony, and also improve CO_2_ removal from the lungs (126, 127). There are 2 main types of NHFV: NHFOV and non-invasive high-frequency percussive ventilation (NHFPV). The former is the most researched one in neonatal medicine.

NHFOV encompasses the delivery of constant distending pressure with pressure amplitude oscillations at a very high rate. Hence, a higher mean airway pressure can be delivered along with adequate carbon dioxide elimination with NHFOV (128). Settings, titration of pressure and amplitude in NHFOV is similar to that in invasive HFOV (129). The interfaces used are similar to the ones used with other NIV devices, but with a few cautionary points. Short binasal prongs and nasal masks are the most favoured interfaces due to reliable tidal volume generation and oscillation transmission (130, 131). CLNT is not preferred for NHFOV as it is shown to increase the work of breathing for the neonate (132).

Multiple observational studies have reported the benefits of NHFOV for neonatal respiratory support. It has been reported that NHFOV use results in decreased treatment failure or need for re-intubation, reduces apnea episodes and improves ventilation without any significant adverse effects (133–135). A recent meta-analysis of 10 RCTs (681 neonates) revealed that NHFOV use is associated with significantly lower need for intubation and IMV [Odds Risk (95% CI): 0.29 (0.2–0.4)] when compared to BiPAP and CPAP (132). Three RCTs included ELGANs in this meta-analysis. This meta-analysis also reported that NHFOV is also associated with improved CO_2_ removal when compared to BiPAP and CPAP [MD (95% CI): −0.46 mm Hg (−0.93 to −0.08)]. These results stayed significant even after adjusting for GA and the use of antenatal corticosteroids (132).

## Temperature and humidity during NIV

The upper respiratory passages perform an important function of heating the ambient air to body temperature and humidifying it to prevent injury to the lower respiratory tract (136). Most of this function is performed in the nasal septum and the conchae (136) which are totally bypassed during IMV by an endotracheal tube. In NIV, the heating and humidification functioning of the upper airway is overwhelmed due to the high gas flow rates delivered by it. This predisposes the neonate to barotrauma, volutrauma and hypothermia along with an increased risk of mortality in preterm neonates due to cold and dry respiratory gases (137). Hence, respiratory gases in IMV need to be warmed up to 37 degrees Celsius and humidified to attain a relative humidity of 100% (136). Similar targets are recommended for most NIV respiratory modalities as their interface and the gas flow rates bypass the innate upper respiratory conditioning function system of the neonate (138). A recent review (23 studies, preterm neonates) revealed that most NIV setups fail to attain the desired inspiratory gas temperature and humidity (138). Moreover, many studies have indicated that the environmental air temperature can have a major effect on the inspiratory gas temperature. The use of radiant warmer can also affect respiratory gas conditioning as indicated by studies comparing the inspiratory gas temperature of neonates nursed in radiant warmer to those nursed in incubators. A decreased inspiratory temperature and humidity of the delivered gas in the radiant warmer group was reported by one study (138).

## Pain management in neonates on NIV

NIV can cause significant discomfort and pain for a preterm neonate. The plausible reasons could be the asynchrony spontaneously breathing neonates, abutment of the nasal interface, and nasal injury (139). Persistent pain in the early neonatal period has been shown to have long-lasting effects, especially on long-term neurodevelopment (140). The first step in attaining adequate neonatal comfort on NIV is choosing an appropriate modality of NIV which is individualized to the neonate's need. This includes the usage of more comfortable devices like HHHFNC compared to CPAP, or interfaces such as CLNT or nasal mask to prevent nasal injury. Also, appropriate synchronization using sNIPPV, NIV-NAVA or NHFV may also decrease the discomfort. Evidence of the efficacy and safety of these modalities have been discussed earlier. A systematic review assessed 45 studies and reported that nasal injury can be reduced through the use of barrier dressings in neonates on NIV [RD (95% CI): −0.12 (−0.20 to −0.04)], and by preferring HHHFNC or nasal mask over short binasal prongs (139). This should be accompanied by timely identification of stress, discomfort or pain in the neonate by the routine use of clinical pain scores. Neonates with ongoing NIV should be treated with non-pharmacological means when the first signs of pain appear (141). This could be followed by the use of analgesic medications if needed. Routine use of analgesia for neonates on NIV is an evolving and controversial subject as the evidence for the same is insufficient. The EUROPAIN cohort study reported that 18% of neonates on IMV and 9% neonates on NIV received analgesia or sedative medications (142). Paracetamol (11%) was the most commonly used medication followed by opioids (6%). Only 45% of the neonates on NIV had pain assessments documented. Dexmedetomidine use in neonates is currently on the rise (143) and preliminary reports suggest that it is safe and efficacious for neonates (144). Overall, there is a gap in the literature on the use of analgesia or sedation in neonates on NIV.

## Conclusions

A holistic approach is needed in the care of preterm neonates to improve their short- and long-term outcomes. NIV is one of the most important components of preterm respiratory care. For most indications, sNIPPV seems to be the most efficacious, and HHHFNC being associated with the least likelihood of nasal injury. Adequately powered RCTs are required to evaluate newer modalities of NIV such as NIV-NAVA and NHFV. Though multiple systematic reviews and meta-analyses have indicated the superiority of one NIV modality over the other in neonates, there is no single NIV modality that universally suits all. Hence, the choice of NIV for a neonate should be individualized based on its efficacy, the disease pathology, resource settings, clinician's familiarity and parental values. Finally, further robust adequately powered trials are needed to compare these modalities in the most vulnerable sub-group of preterm neonates, the ELGAN population.
